# Supplementary material for: Area under the ROC Curve has the most consistent evaluation for binary classification
Source: PLoS One. 2024 Dec 23;19(12):e0316019. doi: 10.1371/journal.pone.0316019 (PMC11666033; doi:10.1371/journal.pone.0316019)

S1 Fig: Model Evaluation for data of different prevalence using Markedness and Diagnostic Odds Ratio.

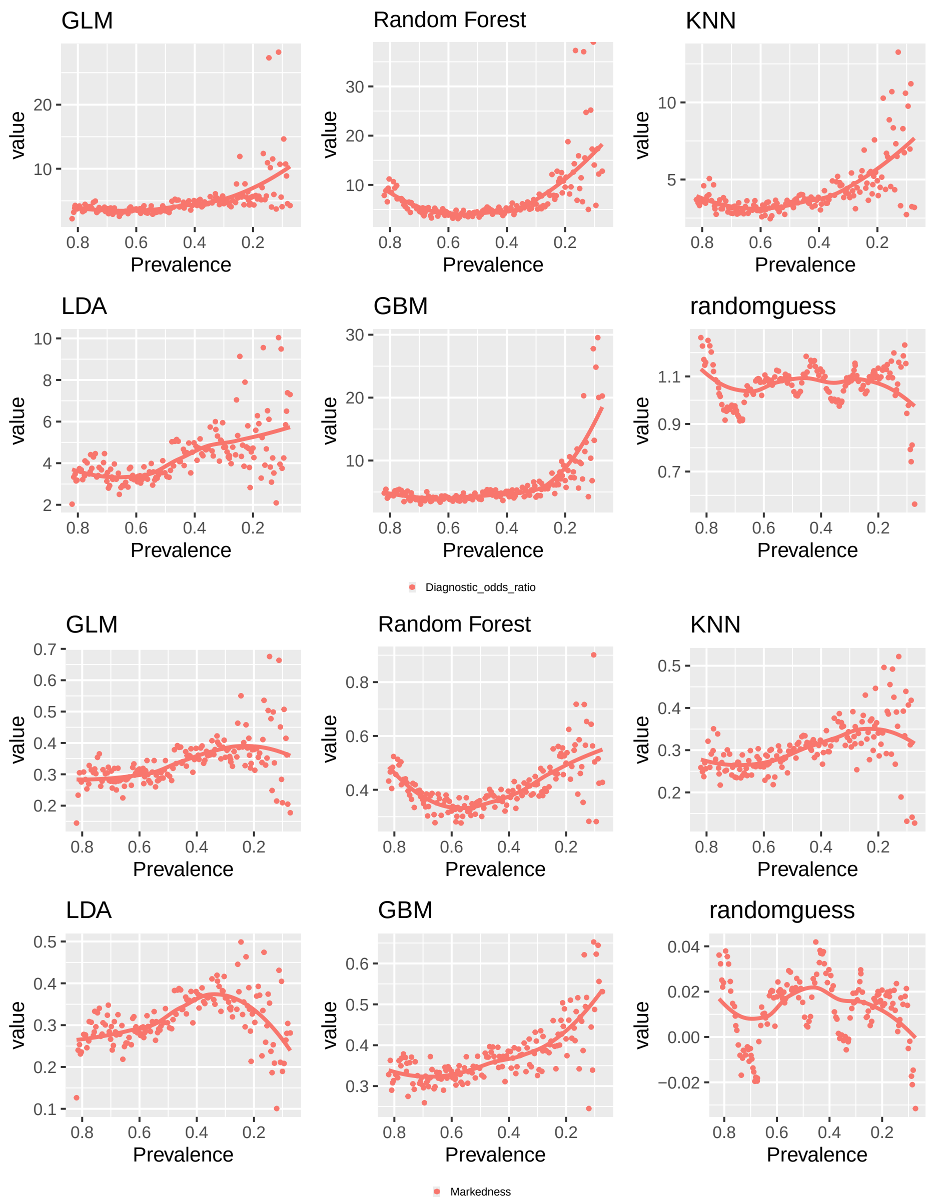

Supplement: S1 Fig — (PDF) [file pone.0316019.s001.pdf]
