# Supplementary material for: Area under the ROC Curve has the most consistent evaluation for binary classification
Source: PLoS One. 2024 Dec 23;19(12):e0316019. doi: 10.1371/journal.pone.0316019 (PMC11666033; doi:10.1371/journal.pone.0316019)

For all models in **S2 Fig**, in certain small regions of the following heat maps, there is change in value for AUC when evaluating using different thresholds, this is just an artifact of plotting. The reason is because there are more than one prevalence level at that point of the x-axis, and they happen to have different AUC. AUC as expected should be completely independent of the specific threshold chosen as it already considers all possible thresholds.

S2 Fig: Model Evaluation for different prevalence and decision threshold for GLM.

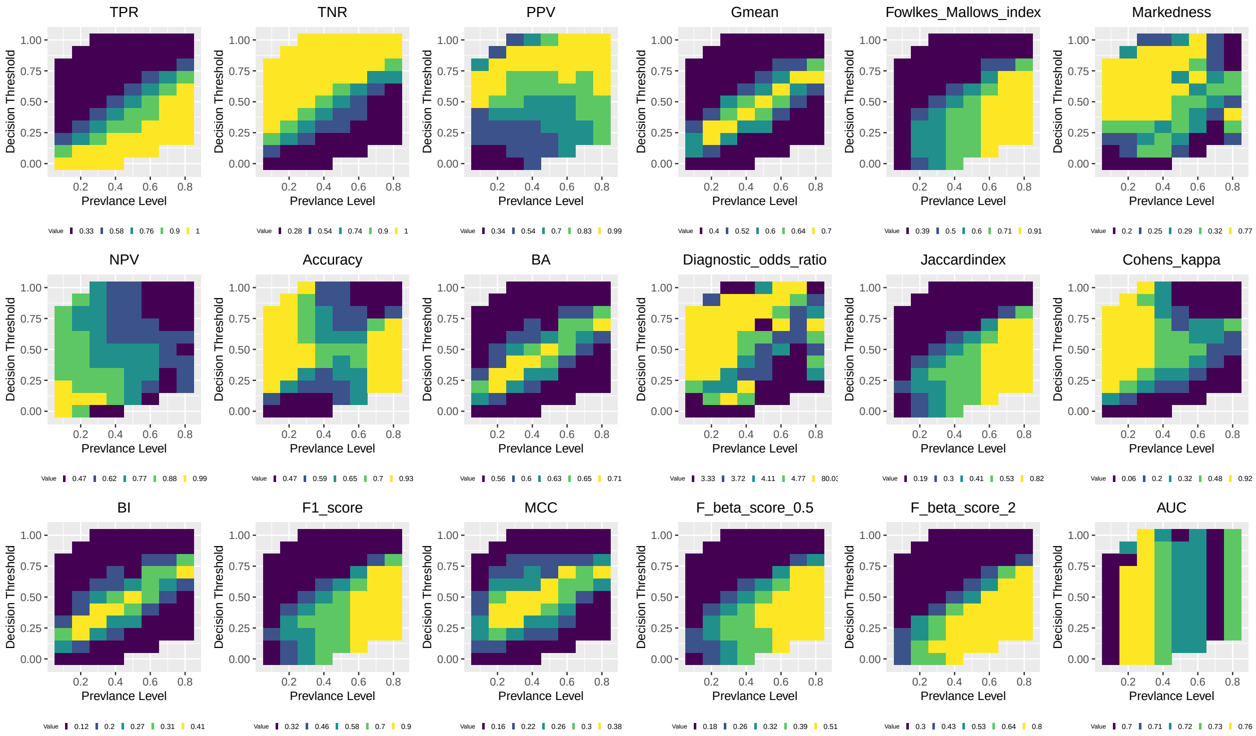

Model Evaluation for different prevalence and decision threshold for KNN

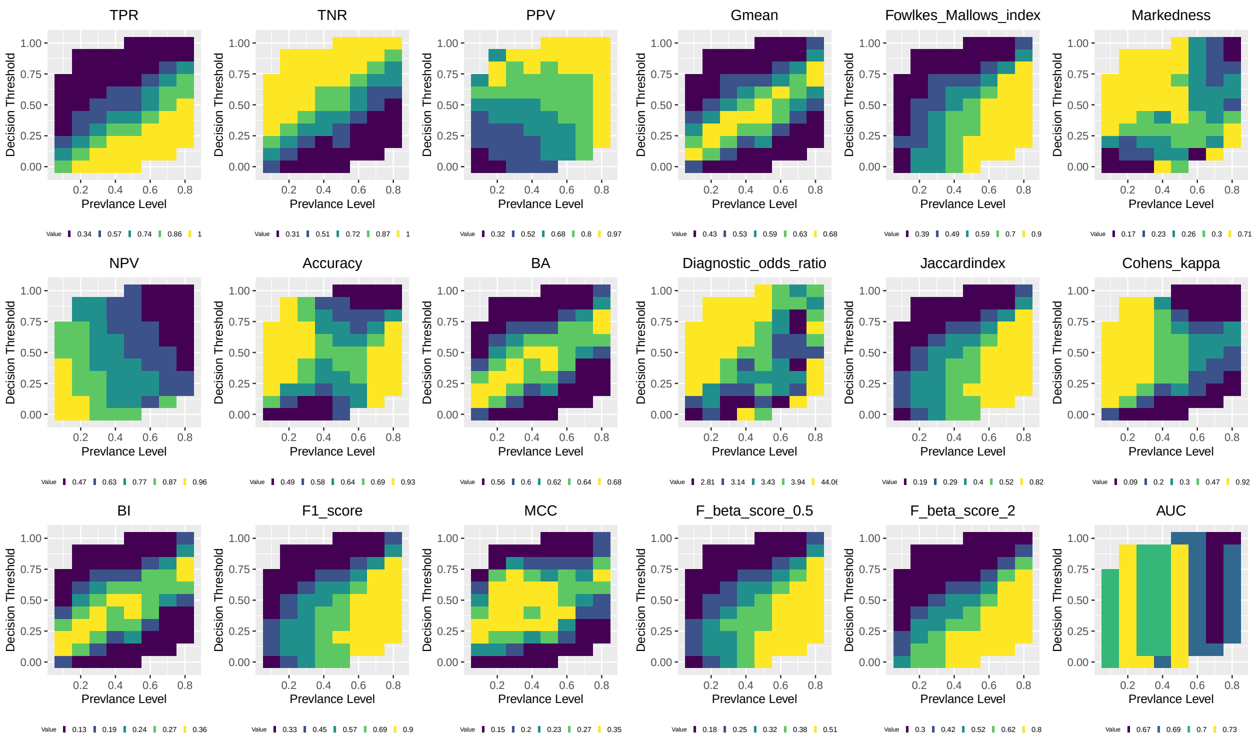

Model Evaluation for different prevalence and decision threshold for LDA

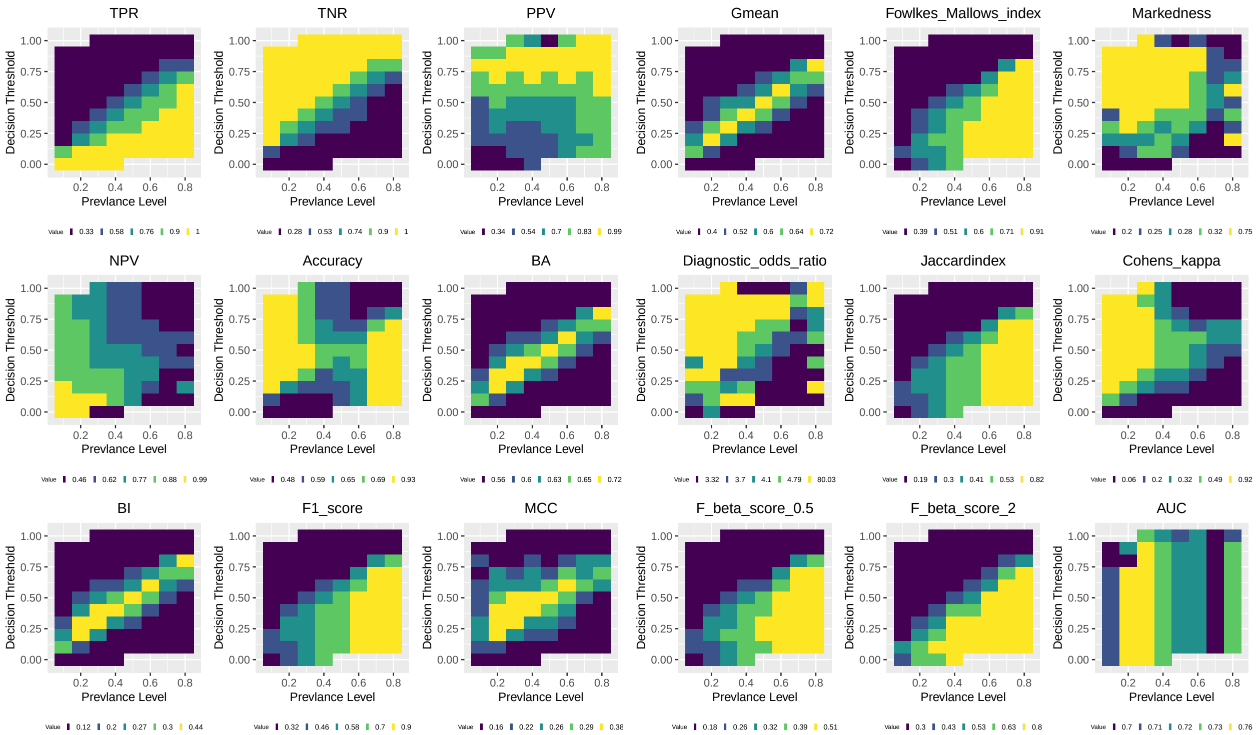

Model Evaluation for different prevalence and decision threshold for GBM

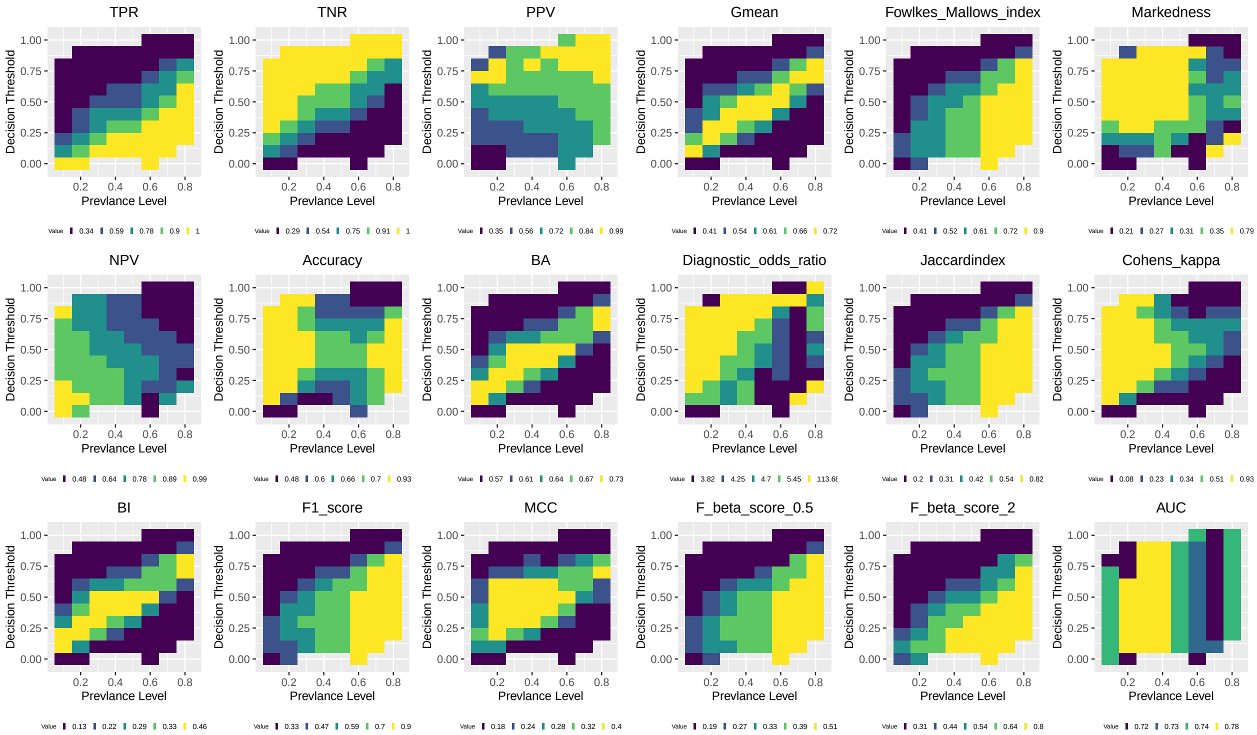

Model Evaluation for different prevalence and decision threshold for Random Guess.

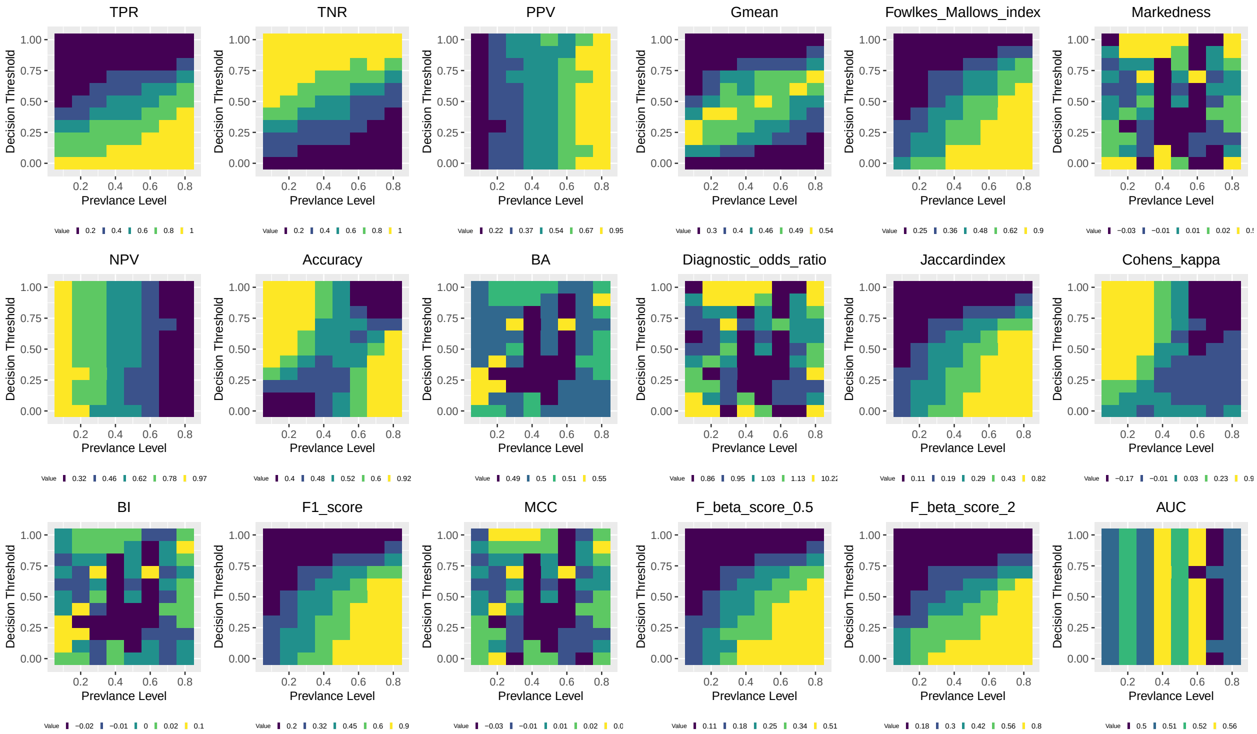

Supplement: S2 Fig — (PDF) [file pone.0316019.s002.pdf]
