# Supplementary material for: Area under the ROC Curve has the most consistent evaluation for binary classification
Source: PLoS One. 2024 Dec 23;19(12):e0316019. doi: 10.1371/journal.pone.0316019 (PMC11666033; doi:10.1371/journal.pone.0316019)

S3 Fig: Correlation Heat Maps for Different Data Samples. Labels for each pane indicates the random number of positive cases being added or dropped from the original data set.

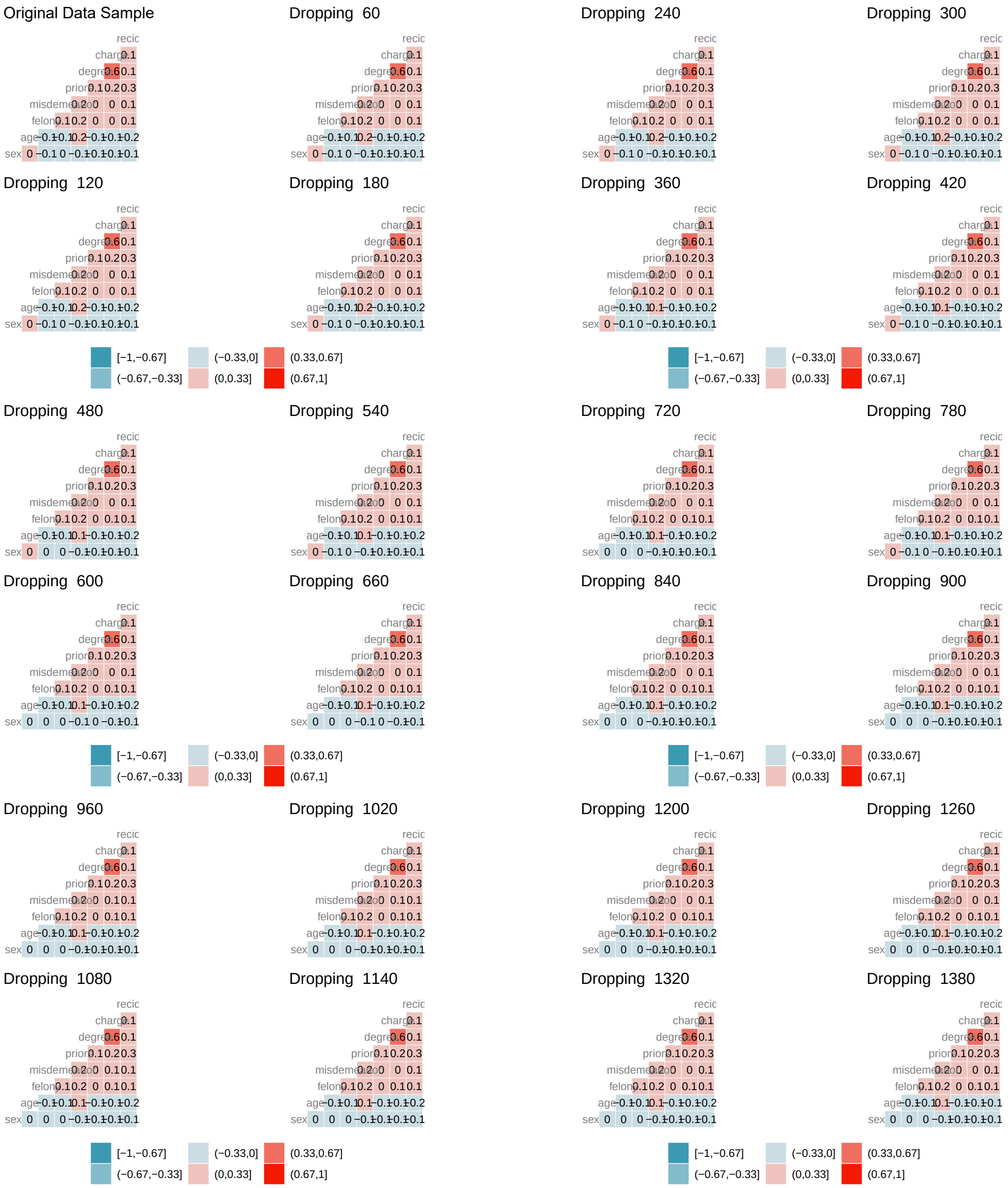

Correlation Heat Maps for Different Data Samples.

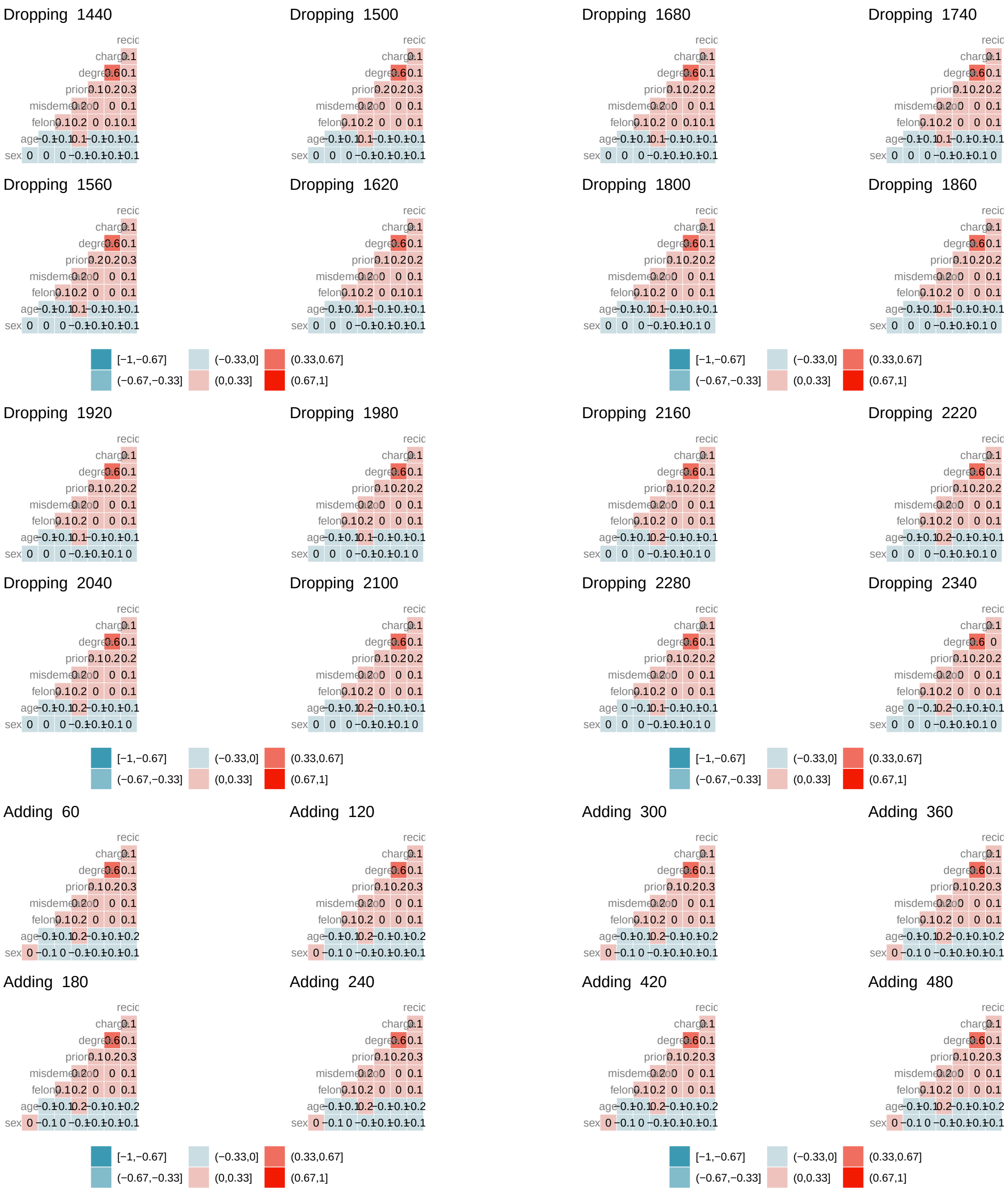

Correlation Heat Maps for Different Data Samples.

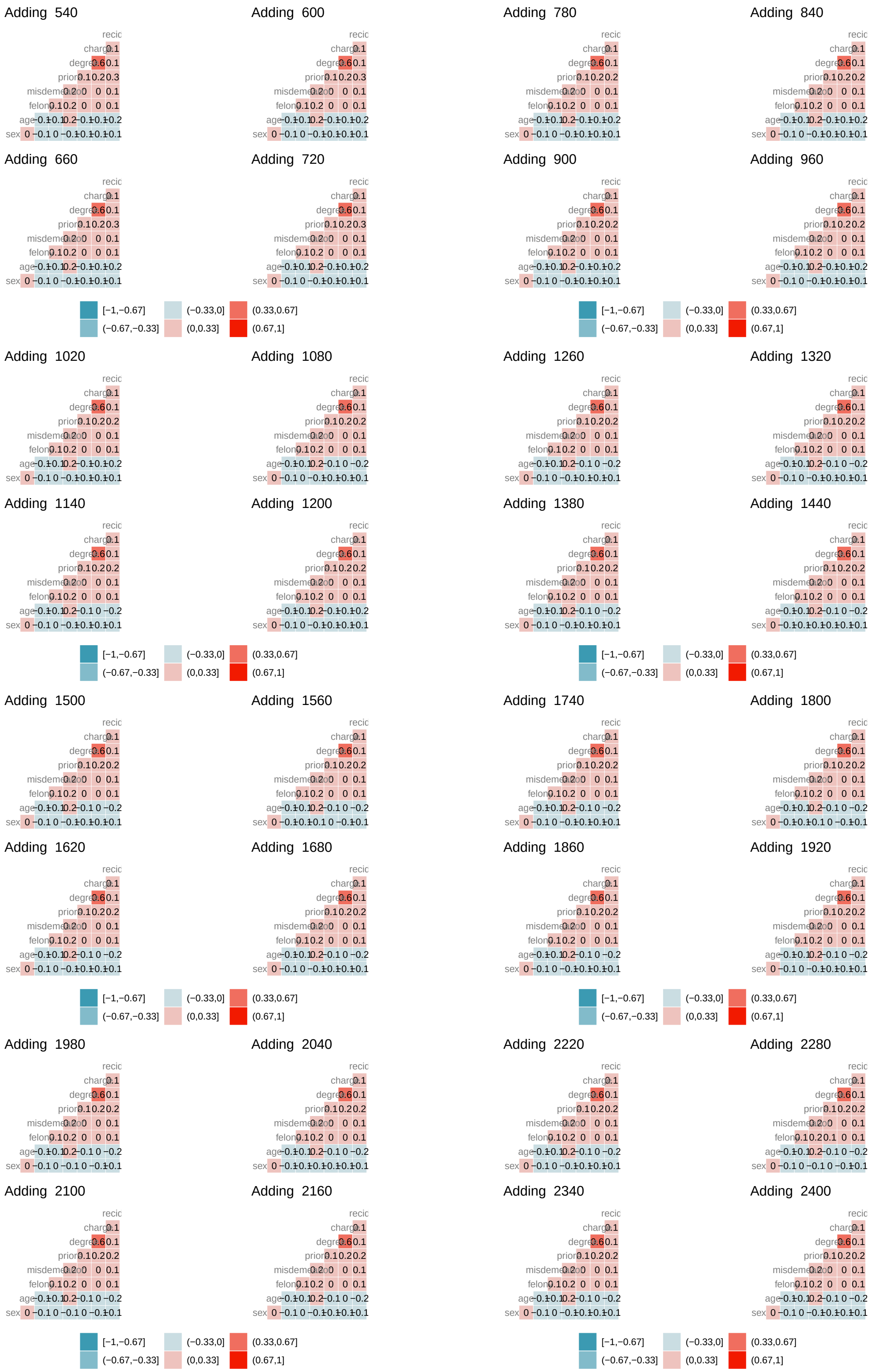

Supplement: S3 Fig — (PDF) [file pone.0316019.s003.pdf]
